# A New Case of Granulomatosis with Polyangiitis Presented with Tolosa–Hunt Syndrome Manifestations

**DOI:** 10.1155/2024/5552402

**Published:** 2024-01-22

**Authors:** Maryam Mohebbi, Shahriar Nafissi, Majid Alikhani

**Affiliations:** ^1^Department of Internal Medicine, School of Medicine, Rheumatology Research Center, Shariati Hospital, Tehran University of Medical Sciences, Tehran, Iran; ^2^Department of Neurology, School of Medicine, Shariati Hospital, Tehran University of Medical Sciences, Tehran, Iran

## Abstract

**Background:**

Tolosa–Hunt syndrome (THS) is a rare disorder involving the orbital and retro-orbital space. The typical symptoms include sensory loss in the trigeminal nerve's distribution, orbital pain, swelling, headaches, and cranial nerve palsies. *Case Presentation*. We report a 40-year-old female who initially presented with biparietal headache, unresponsive to medication, which then led to ophthalmoplegia and orbital pain. Serological findings demonstrated positive CANCA-PR3. She was initially treated with 1 g pulse methylprednisolone for three days. Based on the rheumatological evaluation and her positive lung nodule, hematuria, dysmorphic red blood cells, and positive antiproteinase 3 classic antineutrophil cytoplasm antibodies (CANCA-PR3) and also based on the diagnostic criteria for granulomatosis with polyangiitis criteria for Wegner disease, her treatment was continued with prednisolone 1 mg/kg and also rituximab at the first and 14^th^ day of treatment.

**Conclusion:**

In our case of THS, we achieved satisfactory improvement in symptoms through the administration of high-dose steroids.

## 1. Background

Tolosa–Hunt syndrome (THS) is a rare neurological condition of unknown etiopathogenesis, characterized by unilateral pain and ipsilateral oculomotor paralysis [1]. A generalized inflammation (of unknown origin) in the area of the cavernous sinus and the sphenoid cleft is what causes this disease. Corticosteroid therapy administered correctly within 72 hours significantly slows down its progression [2]. We report a female patient who presented with ophthalmoplegia, headache, and orbital pain and was diagnosed as a case of THS.

## 2. Case Presentation

The patient was a 40-year-old married female, G2P1L1 with a previous medical history of abortion at the 13^th^ week, and also a normal vaginal delivery. Also, the patient had no history of rheumatological diseases. Other than her last coronavirus disease (COVID-19) second booster dose vaccination last year (Sinopharm BBIBP-CorV), she had no other recent vaccinations or infections. She developed coryza symptoms one month before her admission, which was diagnosed as a viral infection and was treated with supportive therapy. However, her dry cough persisted, and she developed a unilateral headache that radiated to her left eye.

She visited a family medicine doctor and was administered diclofenac, ibuprofen, and acetaminophen, which had relatively relieved her headache, but her eye pain persisted. She then visited an internal medicine specialist, who prescribed ketorolac 30 mg intramuscular. However, six hours after the administration of ketorolac, she developed diplopia and her eye pain remained. Therefore, she was admitted to our center for further evaluation.

On her admission, she had diplopia and ptosis in her left eye. She also had a headache, which was unilateral, parietal, nonpulsatile, persistent, localized at her left eye, and unresponsive to medication from one week before her admission. Her vital signs were stable, Glasgow coma scale of 15, and had no significant past medical or drug history. Her physical examination demonstrated left-sided ptosis and limitation of motion, midsize, and reactive pupils, without any dysarthria or fixation preference. She had no significant findings in her skin or joints, and all deep tendon reflexes and other neurological examinations were unremarkable.

Initially, she was visited by an ophthalmologist, who referred her to a neurologist due to no significant findings in her eye examination. During this period, she developed bilateral ptosis and her painful ophthalmoplegia persisted.

**Figure 1:**
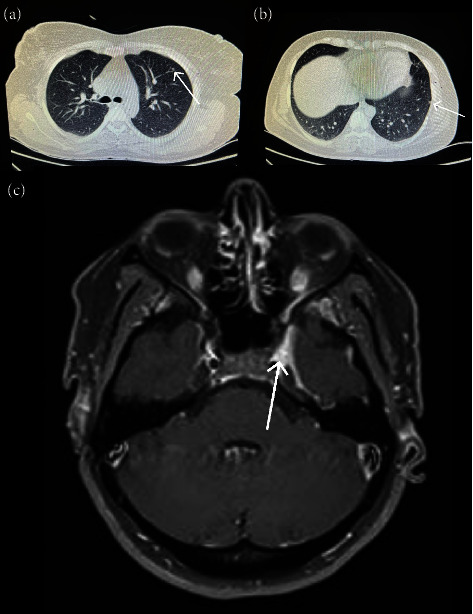
(a, b) Computed tomography scan of lung demonstrating lung nodule; (c) magnetic resonance imaging demonstrating soft tissue enhancement in the left cavernous sinus and superior orbital fissure, with no pressure effect on the carotid artery, in favor of Tolosa–Hunt syndrome.

Brain and orbital magnetic resonance imaging (MRI) with diffusion-weighted imaging and with and without gadolinium demonstrated no abnormalities. Her spiral chest computed tomography (CT) was unremarkable and only demonstrated fibro glandular tissue in her right breast and also two small pulmonary nodules (3 and 4 mm) in her left upper lung (Figure 1).

Cerebrospinal fluid cytological results were acellular, and her viral markers were nonreactive. Other evaluations, including blood, renal, thyroid, and liver function tests, whole blood count, and venous blood gas levels, were unremarkable and are presented in Table 1.

**Figure 2:**
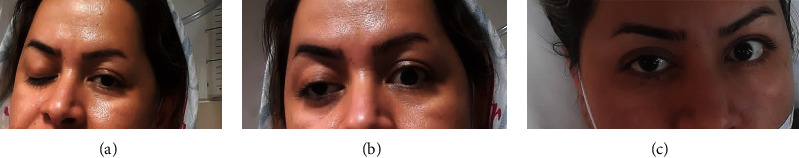
Ptosis of left eye on admission (a), five days after treatment (b), and 21 days after treatment (c).

She was diagnosed with THS based on her ophthalmoplegia, unilateral headache, and orbital pain, along with the MRI findings. The serological findings demonstrated positive classic antineutrophil cytoplasm antibodies-proteinase 3 (CANCA-PR3). She was initially treated with 1 g pulse methylprednisolone for three days. Based on rheumatological evaluation and her positive lung nodule, hematuria and dysmorphic red blood cells (RBCs), and positive CANCA-PR3 and also based on granulomatosis with polyangiitis (GPA) criteria, her treatment was continued with prednisolone 1 mg/kg and also rituximab at the first and 14^th^ day of treatment.  Figure 2 demonstrates the patients' ptosis on admission and during treatment.

## 3. Discussion

Tolosa–Hunt syndrome is a painful ophthalmoplegia brought on by cavernous sinus inflammation that is not specific. The nosology of this syndrome and its existence have both been debated throughout the years [1]. The first patient with this syndrome was described by Tolosa in 1954, who presented with left orbital pain, ipsilateral progressive vision loss, total left ophthalmoplegia, and diminished sensation over the first division of the trigeminal nerve. Hunt described this clinical entity in 1961, seven years after it first appeared, based on six patients [3]. Our patient was diagnosed with THS based on her ophthalmoplegia, headache, and orbital pain. The serological findings demonstrated positive CANCA-PR3. She was initially treated with 1 g pulse methylprednisolone for three days. Based on rheumatological evaluation and her positive lung nodule, hematuria and dysmorphic RBC, and positive CANCA-PR3 and also based on 2022 American College of Rheumatology/European Alliance of Associations for Rheumatology classification criteria for GPA criteria [4] for granulomatosis with polyangiitis disease, her treatment was continued with prednisolone 1 mg/kg and also rituximab at the first and 14^th^ day of treatment.

Controversy exists regarding the pathophysiological and etiology of THS. After ruling out other causes of painful ophthalmoplegia, this diagnosis of exclusion is still used [5, 6]. Hunt [6, 7] has established the following basic clinical standards: one or more episodes of unilateral orbital pain lasting for weeks without treatment; paresis of the third, fourth, or sixth cranial nerve; detection of granulomas by MRI or by biopsy; paresis coupled with unexpected onset of eye pain or pain within two weeks of ocular dysfunction; pain and paresis disappearing within 72 hours if treatment with corticosteroids is sufficient; and other etiologies have been excluded by appropriate investigations.

Patients range in age from 4 to 75 [8], but THS can affect anyone at any age, regardless of sex. Although it is typically unilateral, cases of bilaterality have been documented [9]. Our patient was a 40-year-old female who arrived with bilateral symptoms. Despite the results of all conducted investigations and the response to treatment, the diagnosis of this syndrome was kept. Our patient did not have any of the common causes of painful ophthalmoplegia that were ruled out by extensive diagnostic tests.

Our case discusses how to properly evaluate patients who have eye pain and ocular nerve palsies, collaborate with ophthalmology, rule out acute infections and cancer, and then take into account the uncommon but treatable THS, also known as idiopathic orbital inflammation syndrome. It is crucial that we rule out other possible causes for similar symptoms because metastasis, lymphoma, aspergillosis, or even mucormycosis may present clinically and radiographically similarly and respond to steroids similarly [10]. If these conditions are not correctly diagnosed, it could be fatal. We were able to reduce the patient's symptoms with corticosteroids, which are the cornerstone of treatment, after ruling out differentials [9].

The efficacy of corticosteroid therapy is suggestive, yet not conclusive. To sustain this diagnosis over the long term, extended monitoring spanning several months becomes imperative. Prednisone is administered orally at a high dosage for a duration of 4 weeks as part of the treatment protocol. Notably, significant improvement is often discernible within the initial 24 hours of initiating treatment [11]. In our case, prednisone at a dosage of 1 mg/kg, along with rituximab administered on the first and 14^th^ day of treatment, resulted in complete and marked regression of the symptoms. During more than six months of follow-up, our patient exhibited no indications of clinical abnormalities. According to Zhang et al. [1], 77.5% of patients achieved complete relief from orbital pain within a week of commencing steroid treatment. The efficacy of corticosteroid therapy is well-documented in various studies [3, 6, 12, 13].

## 4. Conclusion

THS, as a rare but debilitating illness, is frequently diagnosed through exclusion. A diagnosis of the syndrome can be made with 95–100% sensitivity using the triad of one or more episodes of unilateral orbital discomfort, paresis of one or more cranial nerves, and granulomas by MRI or biopsy. Even though the traditional triad was not evident when she was diagnosed, our treatment with high-dose steroids resulted in a notable improvement in symptoms and the prevention of remissions. Therefore, it is critical for doctors to identify THS in their differential diagnosis of painful ophthalmoplegia and to treat the illness quickly to reduce the patient's symptom progression and to enhance treatment.

## Figures and Tables

**Table 1:** On admission laboratory results of a 40-year-old patient with Tolosa–Hunt syndrome.

Test (value)	Result	Reference value	Interpretation
White blood cell count (×10^9^/L)	8.62	4.5–11	Normal
Hemoglobin (g/L)	14.9	13.2–17.3	Normal
Platelet count (×10^*∗*^3/*μ*l)	351	150–450	Normal
Blood urea nitrogen (mg/dL)	9.5	7–20	Normal
Creatinine (mg/dl)	1.04	0.5–1.4	Normal
Lactate dehydrogenase (IU/L)	302	0–500	Normal
Troponin	Negative	Negative	Normal
Blood sugar (mg/dL)	99	100–125	Normal
Prothrombin time (sec)	11.87	10–13	Normal
Partial thromboplastin time (sec)	29	25–35	Normal
international normalized ratio	1.02	<1.1	Normal
Sodium (mEq/L)	140	135–145	Normal
Potassium (mEq/L)	4.10	3.5–5.3	Normal
Calcium	9	8.5–10	Normal
Aspartate aminotransferase (U/L)	19	5–40	Normal
Alanine aminotransferase (U/L)	9	5–40	Normal
Alkaline phosphatase (IU/L)	220	44–147	Normal
Total protein (g/dl)	7.5	6.6–7.8	Normal
Albumin (g/dl)	4.98	3.8–5.1	Normal
T3 (nmol/L)	0.93	1.2–2.8	Normal
T4 (*μ*g/dL)	9.07	5.0–12.0	Normal
Thyroid-stimulating hormone (mU/l)	3.44	0.3–4	Normal
Erythrocyte sedimentation rate (mm/hr)	7.9	<20	Normal
C-reactive-protein (mg/L)	5	<6	Normal
Complement component 3 (mg/dL)	122	70–196	Normal
Complement component 4 (mg/dL)	16	10–40	Normal
Rheumatoid factor (IU/mL)	5	<15	Normal
Antinuclear cytoplasm antibody-proteinase 3	33.7	<10	High
Perinuclear antineutrophil cytoplasmic antibodies-myeloperoxidase	22.8	<10	High
Anti-Sjögren's syndrome type B-LA (IU/ml)	16.7	<7	High
Anti-Sjögren's syndrome type A-RO (IU/ml)	11.7	<7	High
Antinuclear antibody (IU/ml)	0.3	<0.1	High
Creatinine kinase (mcg/L)	63	10–120	Normal
Human T-lymphotropic virus (serum)	Negative	Negative	Normal
Carbon dioxide (mEq/L)	37.7	23–30	Normal
Bicarbonate (mEq/L)	20.3	22–28	Normal
Angiotensin converting enzyme (nmol/mL/min)	<2.4	<40	Normal
Anticardiolipin IgG (U/mL)	9.4	<15	Normal
Anticardiolipin IgM (U/mL)	13.5	12.5–20	Normal
Hepatitis B virus surface antigen	Negative	Negative	Normal
Hepatitis B virus core antibody	0.03	<1	Normal
Hepatitis C antibody	Negative	Negative	Normal
Human immunodeficiency virus antibody	Negative	Negative	Normal
SARS-CoV-2 (polymerase chain reaction)	Negative	Negative	Normal
Urinalysis	Blood 1+ (red blood cells: 8–10, dysmorphic 20%)	—	Hematuria
Purified protein derivative test	<1 cm	<1.5 cm	Negative
Cerebrospinal fluid	Protein: 31; lactate dehydrogenase: 24; sugar: 58; culture: negative; cell count: red blood cells: not seen, white blood cells: 1	—	Normal, acellular

## Data Availability

The data used to support the findings of the study are available from the corresponding author upon request.

## Abbreviations

CANCA-PR3: Classic antineutrophil cytoplasm antibodies-proteinase 3
CT: Computed tomography
ESR: Erythrocyte sedimentation rate
GPA: Granulomatosis with polyangiitis
MRI: Magnetic resonance imaging
RBC: Red blood cell
THS: Tolosa–Hunt syndrome.
